# Supplementary figures and images for: Monosynaptic trans-collicular pathways link mouse whisker circuits to integrate somatosensory and motor cortical signals
Source: PLoS Biol. 2023 May 19;21(5):e3002126. doi: 10.1371/journal.pbio.3002126 (PMC10234540; doi:10.1371/journal.pbio.3002126)

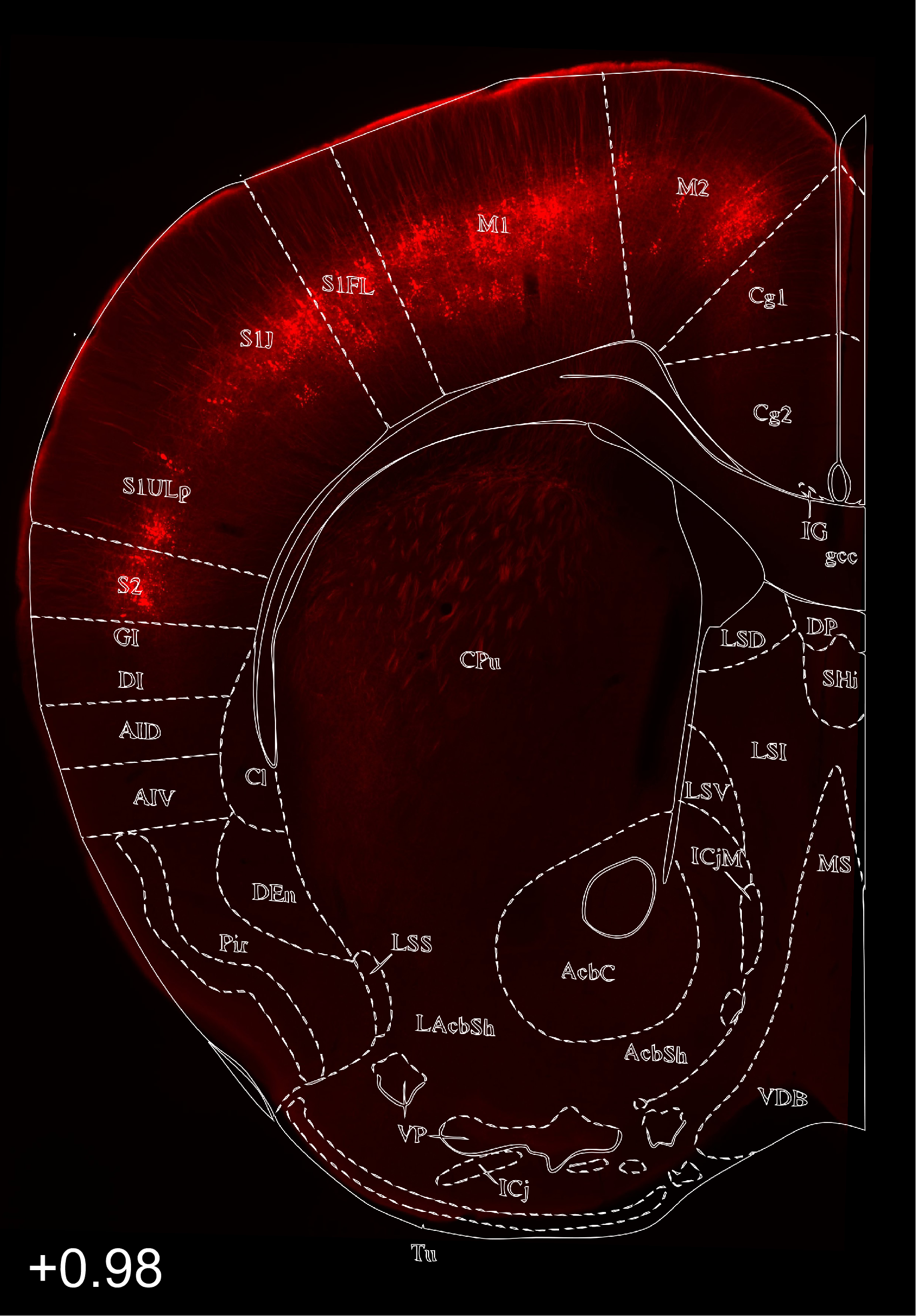

Supplement: S1 Fig — Related to Fig 3. (A) Retrograde mCherry-labeled LSC-projecting neurons (red) in the MC extend across M1 and M2. (B) Higher magnification confocal image showing MC LSC-projecting neurons (red) in the motor cortex from pia to wm. LSC, lateral SC; MC, motor cortex; wm, white matter. (TIF) [file pbio.3002126.s001.tif]

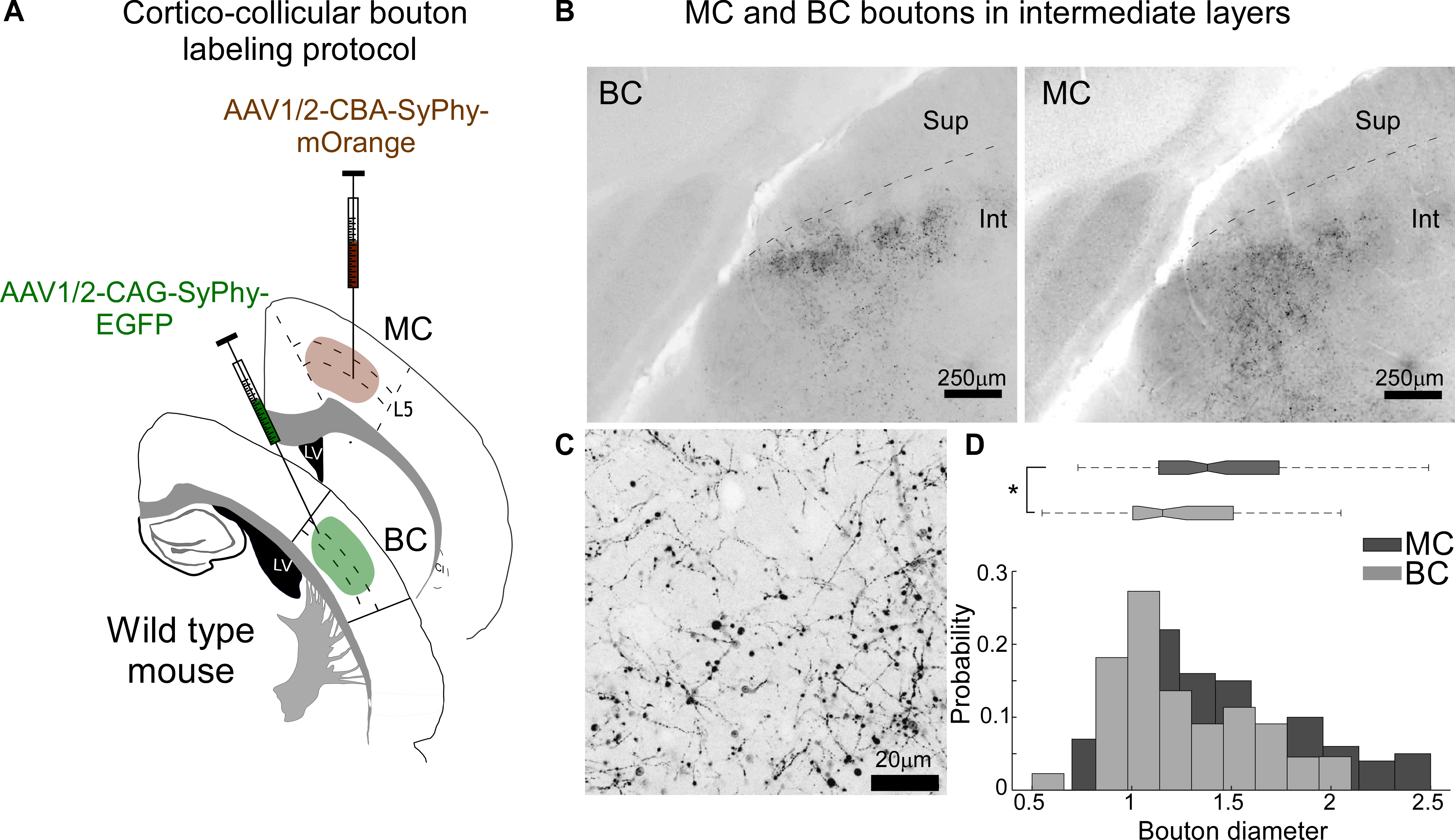

Supplement: S2 Fig — Related to Fig 3. (A) Schematic of dual injections of AAVs encoding for synapse-specific fluorescent fusion proteins (Synaptophysin-mOrange, Synaptophysin-EGFP; [1,2]) to label MC (mOrange) and BC (EYFP) boutons in SC. (B) Example confocal images of coronal SC slices with fluorescently labeled BC (left, synaptophysin-EGFP) and MC (right, synaptophysin-mOrange) boutons. (C) Example of higher magnification confocal image showing BC boutons in the intermediate layers of SC. (D) Normalized distribution of MC and BC bouton diameters in SC. Boxplots of bouton diameters, median (line in box), IQR (first to third quartile, boxes) (BC = 1.15 μm, n = 100; MC = 1.38 μm, n = 44) and IQRs (BC = 0.50 μm; MC = 0.60 μm). * represents p < 0.01; B: Wilcoxon rank sum; exact p-values in S1 Table. The data for S2D Fig can be found at: https://doi.org/10.11588/data/DNOSZG. BC, barrel cortex; Int, intermediate layer; IQR, interquartile range; MC, motor cortex; SC, superior colliculus; Sup, superficial layer. (TIF) [file pbio.3002126.s002.tif]

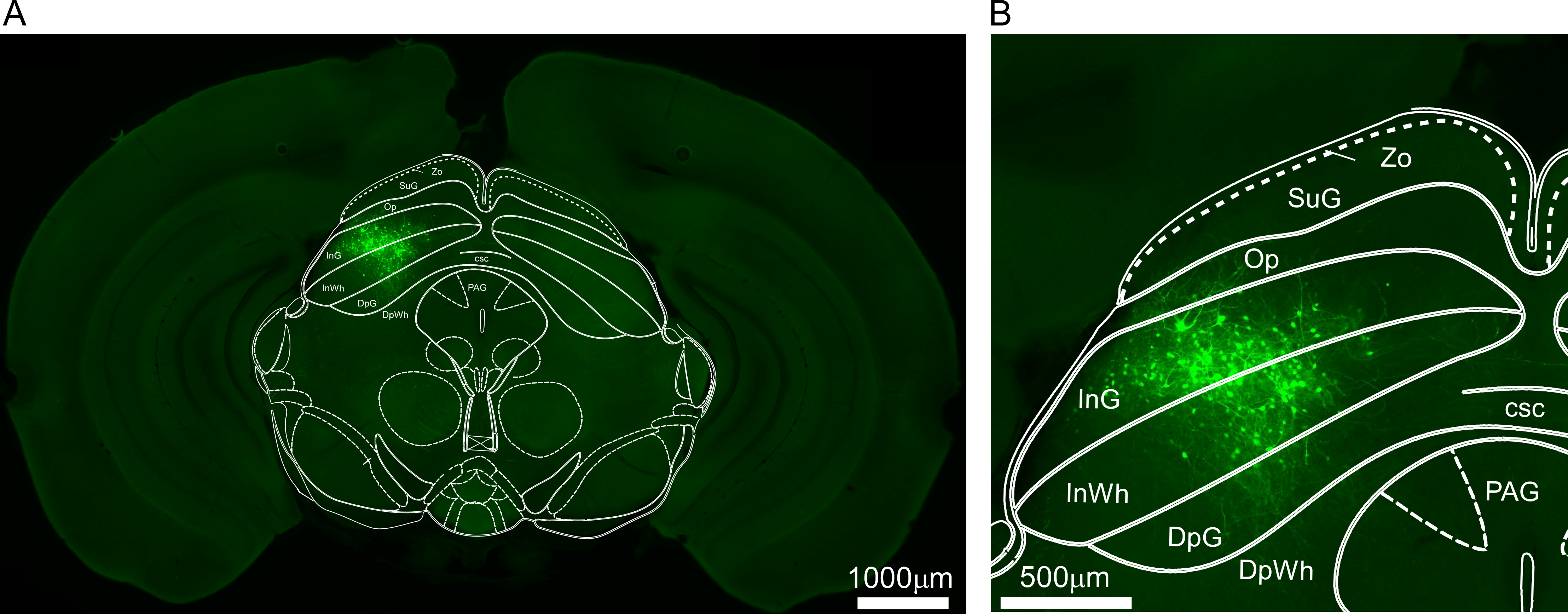

Supplement: S3 Fig — Related to Fig 4. (A) SC coronal slice showing trans-synaptically EGFP-labeled MC-RNs in the intermediate layers of SC with overlaid anatomical borders from Paxinos mouse brain atlas [3]. (B) Same as (A) at higher magnification. csc, commissure of the superior colliculus; DpG, Deep gray layer; DpWh deep white layer; InG, Intermediate gray layer; InWh, intermediate white layer; MC, motor cortex; Op, optic nerve of the superior colliculus; PAG, periaqueductal gray; RN, recipient neuron; SC, superior colliculus; SuG, superficial gray layer; Zo, Zonal layer. (TIF) [file pbio.3002126.s003.tif]

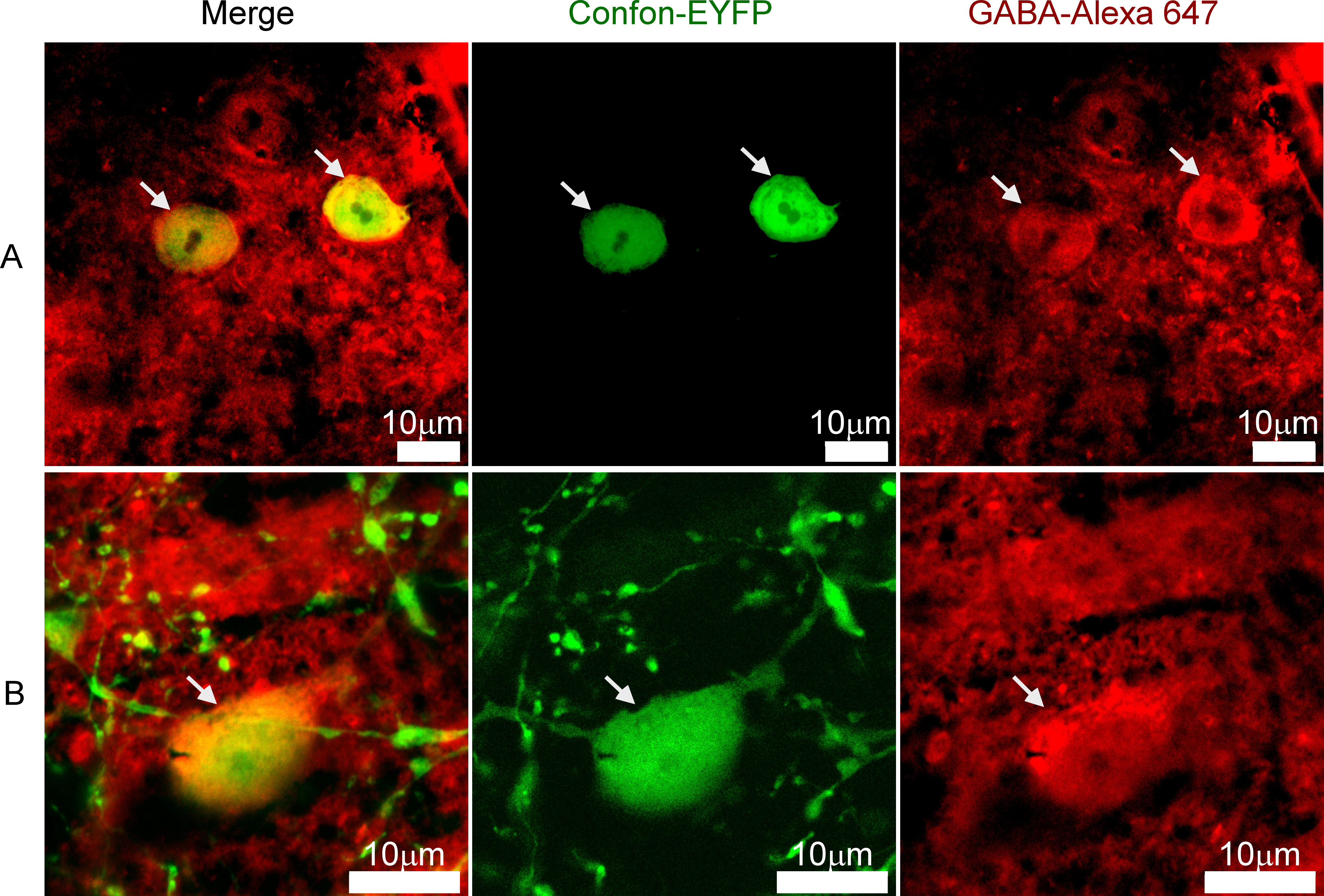

Supplement: S4 Fig — Related to Fig 5. Example experiment in which MC-iRNs were labeled by injecting AAV1-Flpo in MC, and AAV8-Con/Fon-EYFP in LSC of GAD-Cre mice. Slices with trans-synaptically labeled iRNs (green, Confon-EYFP) were counterstained against GABA (red, immunostaining with Alexa 647; see Materials and methods). All inspected iRNs were GABA positive. (A) Example confocal image showing iRNs (green) and GABA immunostain (red). Left: Overlay of red and green channel, showing GABA-positive iRNs in yellow (arrows). Middle: Green channel, showing iRNs (green). Right: Red channel, showing GABA-positive somata (red). (B) Same as in (A) for a different iRN at higher magnification. iRN, inhibitory RN; LSC, lateral SC; MC, motor cortex. (TIF) [file pbio.3002126.s004.tif]

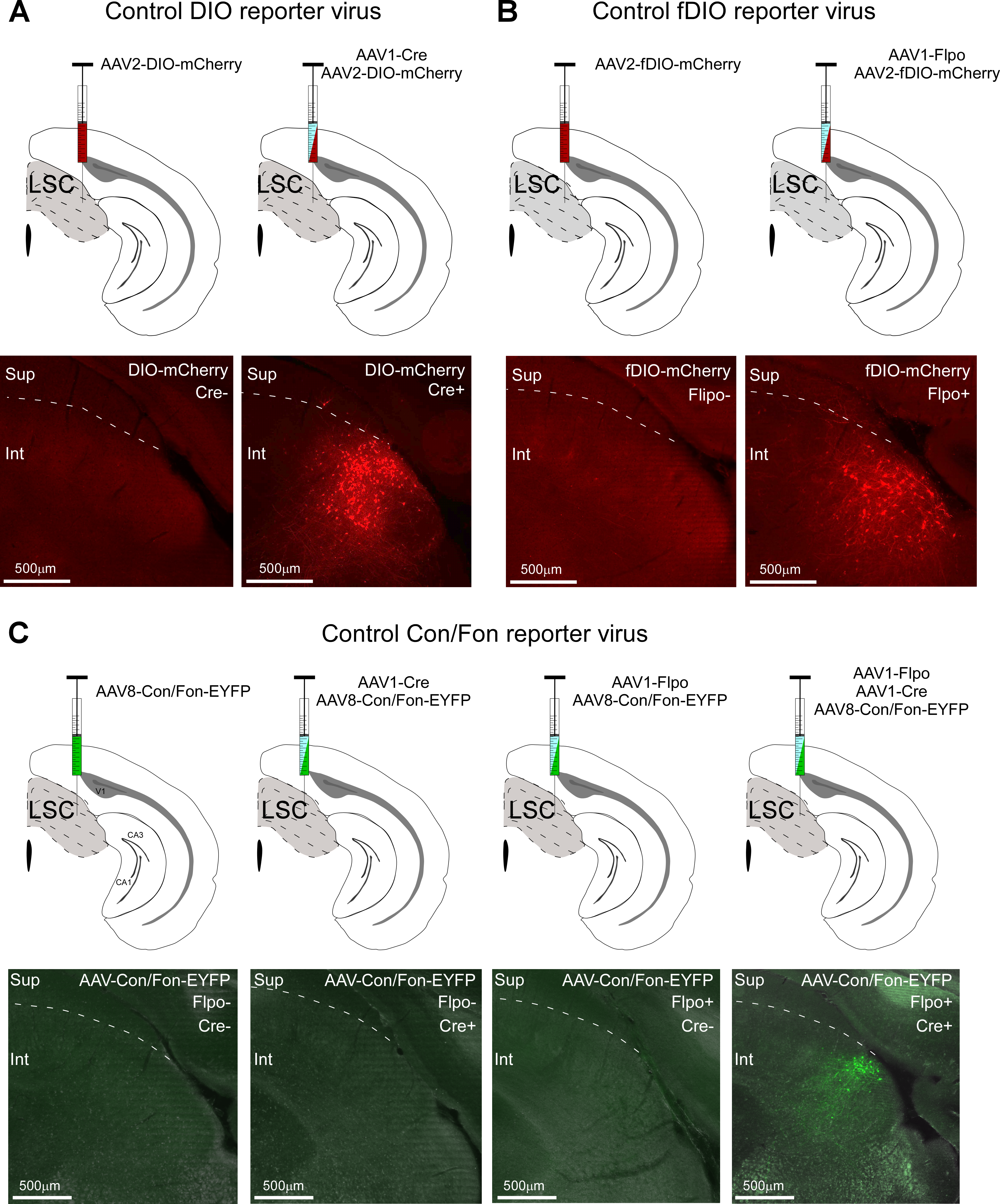

Supplement: S5 Fig — Related to Figs 4–6. (A) Upper row: Conditional expression of the reporter DIO was tested by injecting AAV2-DIO-mCherry either alone (left) or in combination with AAV1-Cre (right) into the LSC. Bottom row. Examples of corresponding fluorescence images of the LSC. Conditional expression was only observed in combination with AAV1-Cre (right, red neurons). No leak expression of the reporter virus was detected. (B) Same as (A) but for Flpo-dependent reporter virus. No leak expression of the reporter virus was detected. (C) Upper row: Conditional expression of the reporter virus AAV8-Con/Fon-EYFP was tested by LSC injections of AAV8-Con/Fon-EYFP either alone or in different combinations with trans-synaptic viruses: AAV1-Cre and AAV1-Flpo. Bottom row: Examples of corresponding fluorescence images of the LSC. Conditional expression was only observed for the last combination AAV8-Con/Fon-EYFP and AAV1-Cre and AAV1-Flpo (green neurons); no leak expression of the reporter virus was detected. (TIF) [file pbio.3002126.s005.tif]

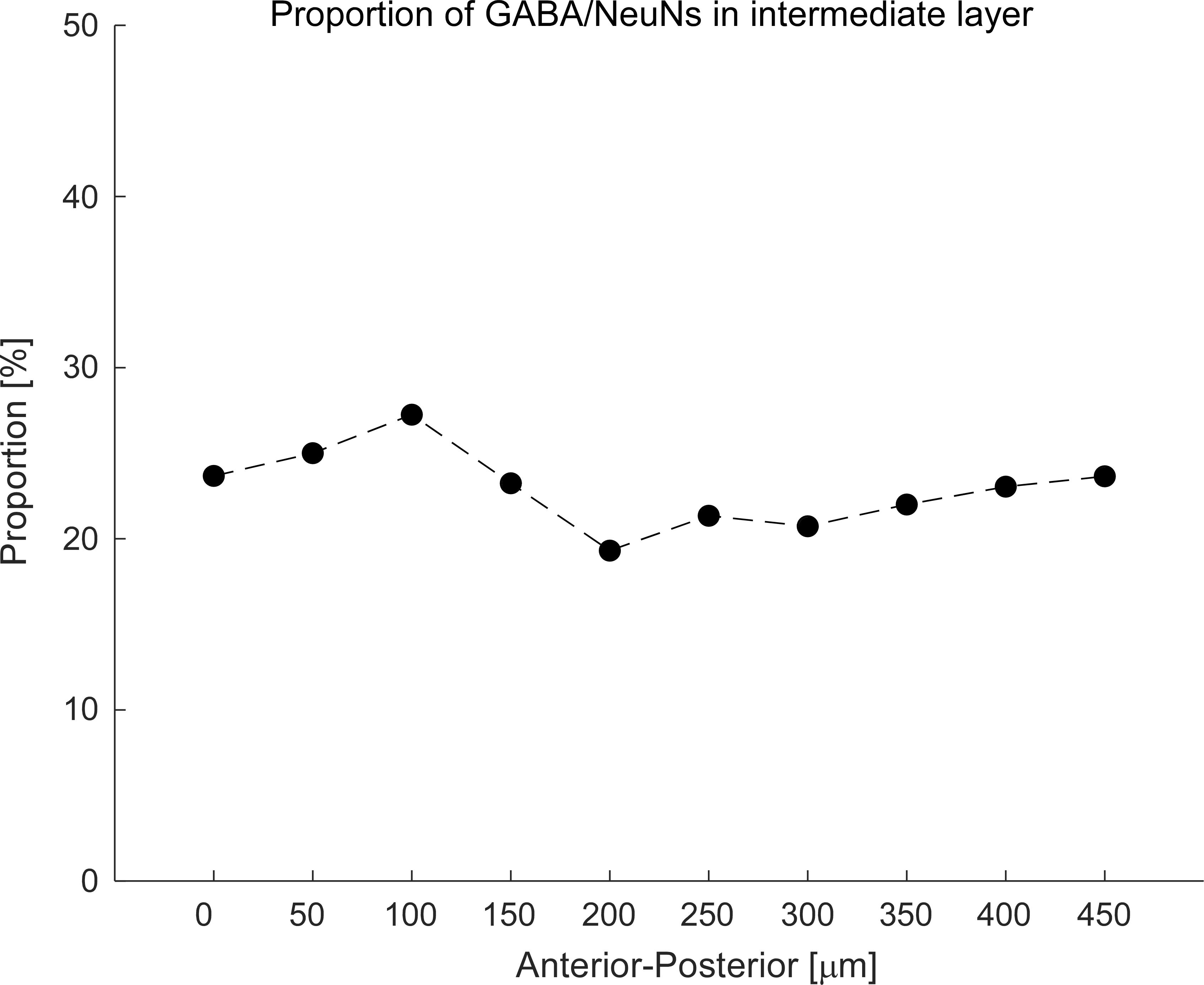

Supplement: S6 Fig — Related to Fig 5. Proportion of GABAergic GAD67-GFP positive neurons with respect to immunostained NeuN neurons in the intermediate layer of the LSC. Using a GAD-GFP mouse line, in which GABAergic neurons are labeled with GFP, neurons were counterstained with NeuN-Alexa 647 (Fig 5). The plot shows the proportion of GABA and NeuN neurons along the rostro-caudal LSC axis. The data for S6 Fig can be found at: https://doi.org/10.11588/data/DNOSZG (TIF) [file pbio.3002126.s006.tif]

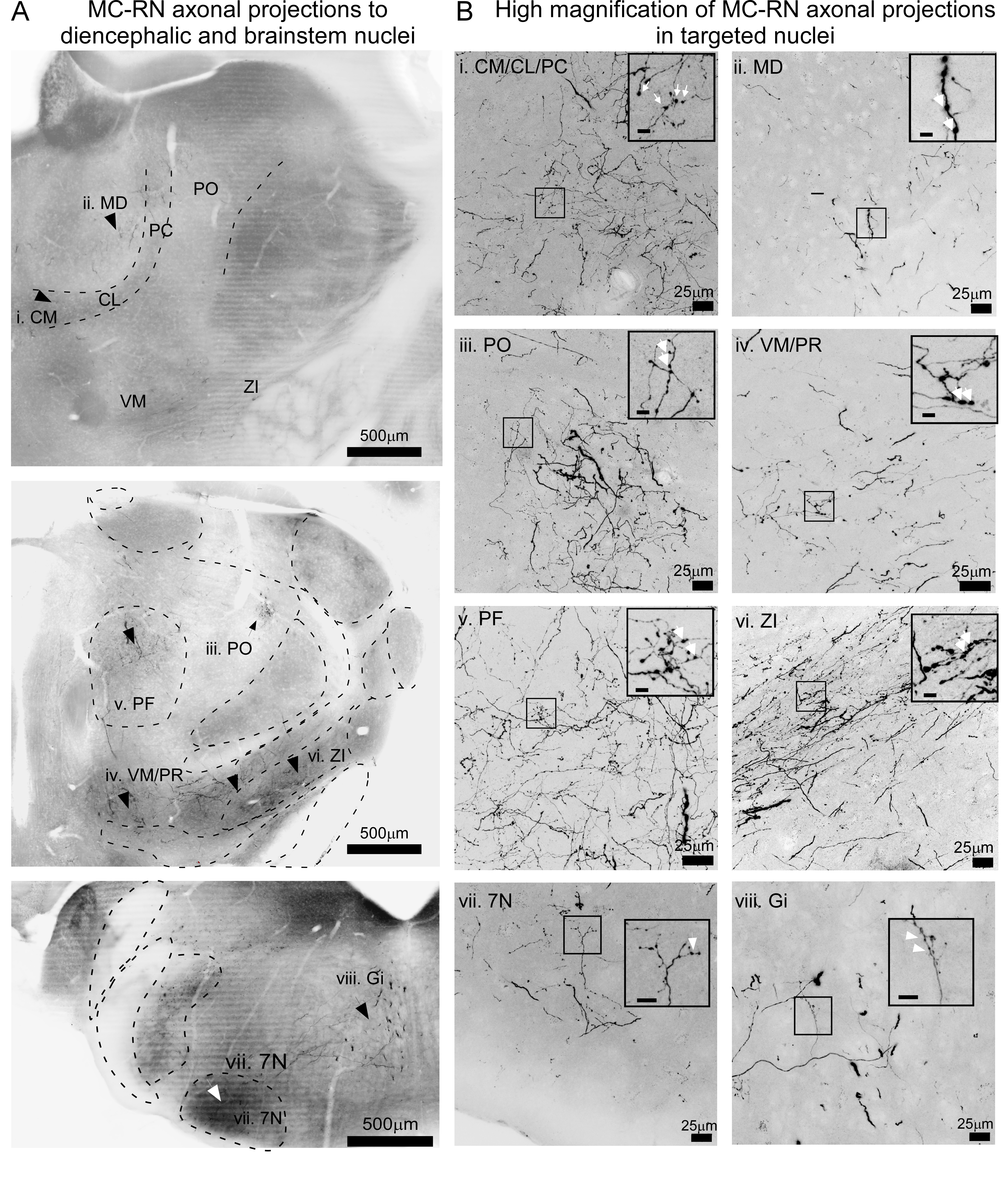

Supplement: S7 Fig — Related to Fig 8. (A) Desaturated and inverted fluorescence images at 2 different rostro-caudal coordinates in diencephalic regions (top and middle) and brainstem (bottom), showing MC-RN axons (black arrowheads) at low magnification. Roman numbers enumerate the nuclei shown in (B). (B) Confocal images show examples of axons in diencephalic nuclei CM/CL/PC MD, PO, VM, PF, and ZI and in brainstem nuclei, Gi and 7N. Roman numbers indicate the nuclei shown in (A). Small square box indicates the region selected for magnification. Insets show a high magnification of axons and varicosities (white arrowheads), scale bar = 5 μm. CL, centrolateral nucleus; CM, centro-medial nucleus; Gi, gigantocellular reticular nucleus; MC, motor cortex; MD, mediodorsal nucleus; PC, precentral; PF, parafascicular nucleus; PO, posterior nucleus; RN, recipient neuron; nucleus VM, ventromedial nucleus; ZI, zona incerta; 7N, Facial nucleus. (TIF) [file pbio.3002126.s007.tif]

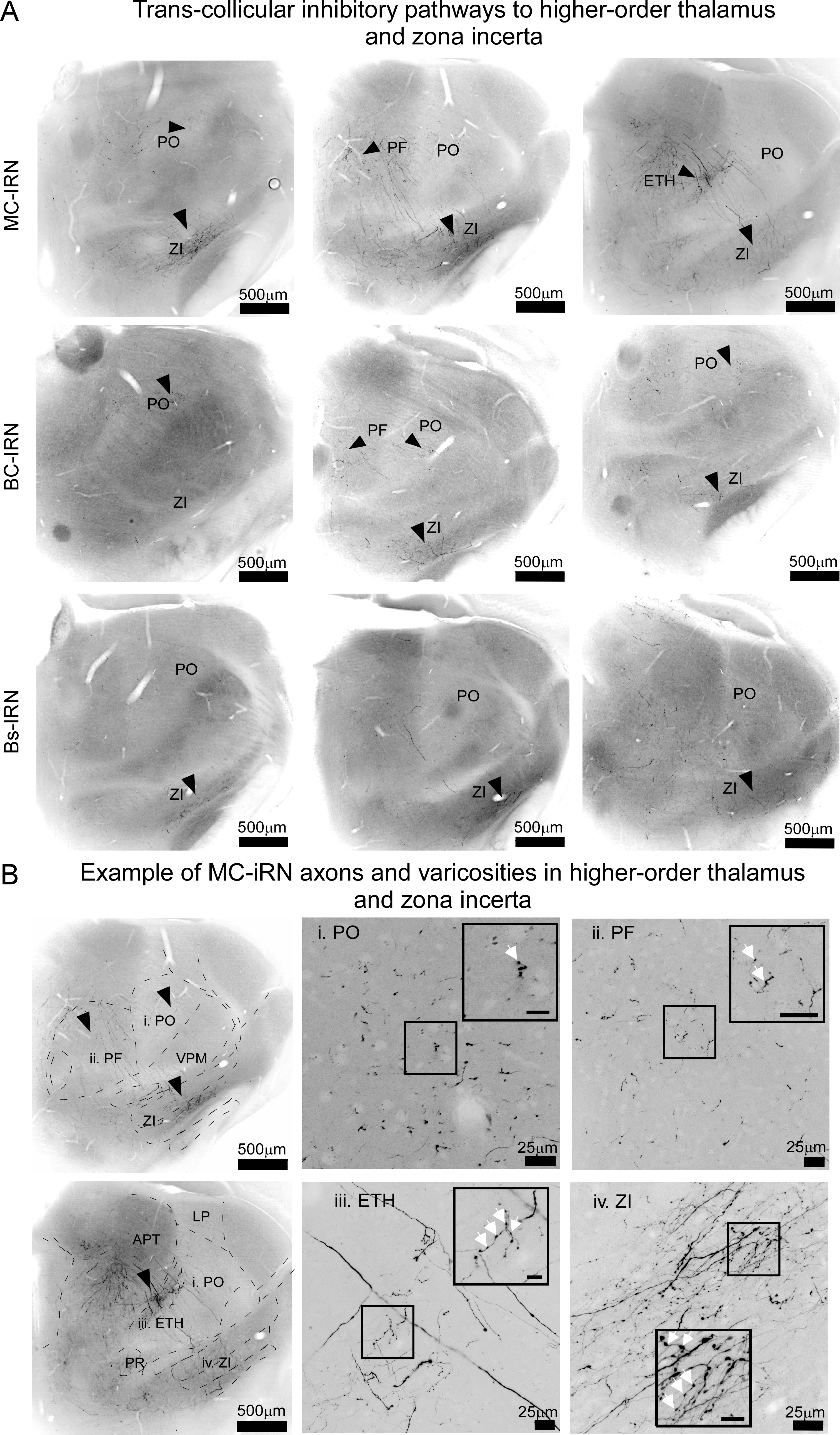

Supplement: S8 Fig — Related to Fig 8. (A) Desaturated and inverted fluorescence images at 3 different rostro-caudal coordinates (from left to right) showing MC-, BC-, and Bs-iRNs outputs (top to bottom). Black arrowheads indicate iRN axons. (B) Left: Exemplary desaturated and inverted fluorescence images at 2 different rostro-caudal coordinates showing MC-iRNs outputs in diencephalic nuclei (top and bottom). Black arrowheads indicate axons. Roman numbers enumerate the nuclei shown in high magnification. ZI, zona incerta. Right: High magnification confocal images show examples of MC-iRN axons in ETH PF, Po, and ZI. Small square box indicates the region selected for magnification Roman numbers indicate the nuclei shown left. Insets show a high magnification of axons and varicosities (white arrowheads), inset scale bar = 10 μm. APT, anterior pretectal area; BC, barrel cortex; Bs, brainstem; ETH, ethmoidal nucleus; iRN, inhibitory RN; LSC, lateral SC; MC, motor cortex; MD, mediodorsal nucleus; Pf, parafascicular nucleus; PO, posterior nucleus; RN, recipient neuron; VM, ventromedial nucleus; ZI, zona incerta. (TIF) [file pbio.3002126.s008.tif]

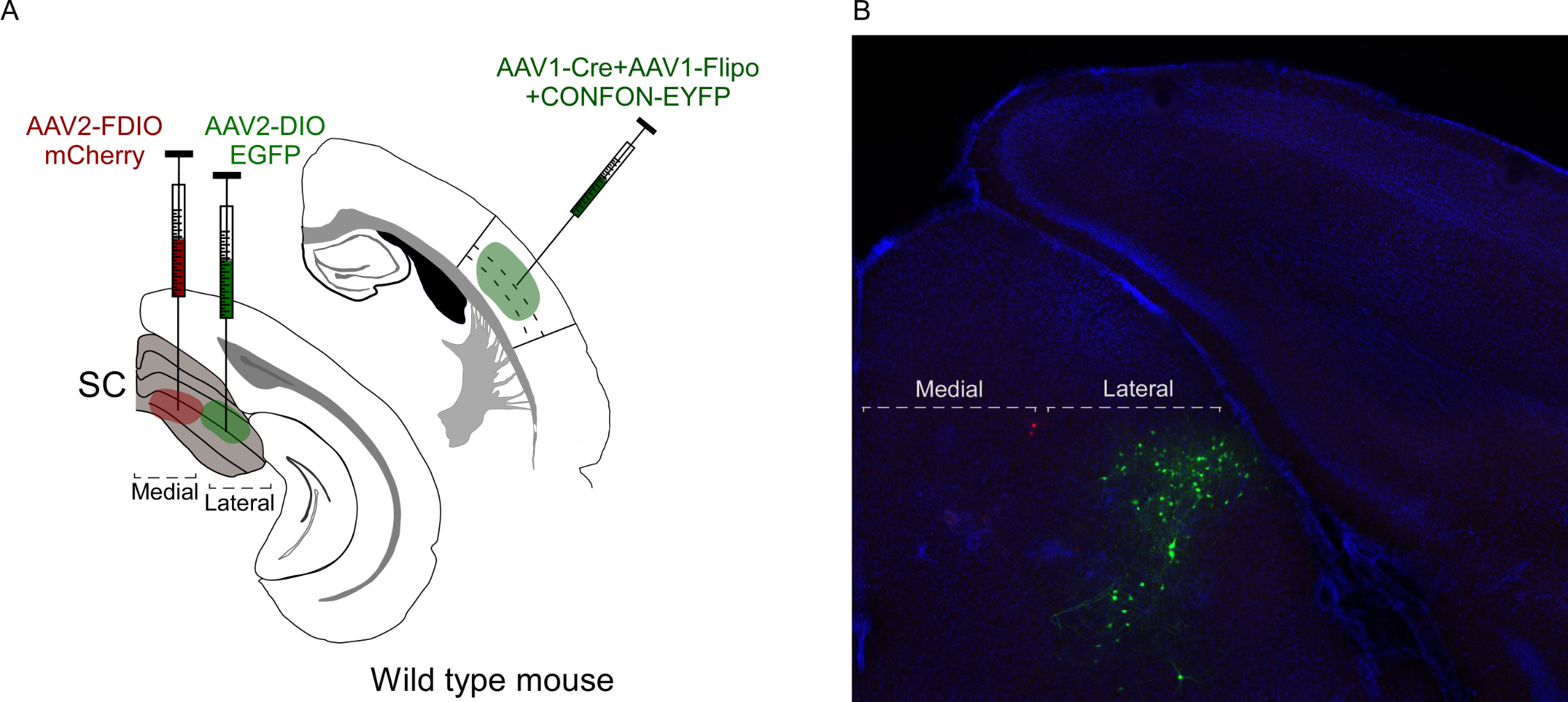

Supplement: S9 Fig — Related to Figs 4, 5 and 6. (A) Experimental schematic of trans-synaptic labeling to validate sufficient spread of the reporter virus to label most of the RN population in the recipient zone in SC. A cocktail of AAV1-Cre + AAV1-Flpo and reporter AAV8-ConFon-EYFP was injected in the barrel cortex. In SC, 2 different reporters fDIO-mCherry and DIO-EGFP were injected into adjacent locations corresponding to the medial and lateral zone of SC, respectively. (B) SC coronal slice showing that BC-RNs were mostly labeled with EGFP in the lateral zone and very few BC-RNs were labeled by mCherry in the medial zone. BC, barrel cortex; RN, recipient neuron; SC, superior colliculus. (TIFF) [file pbio.3002126.s009.tiff]

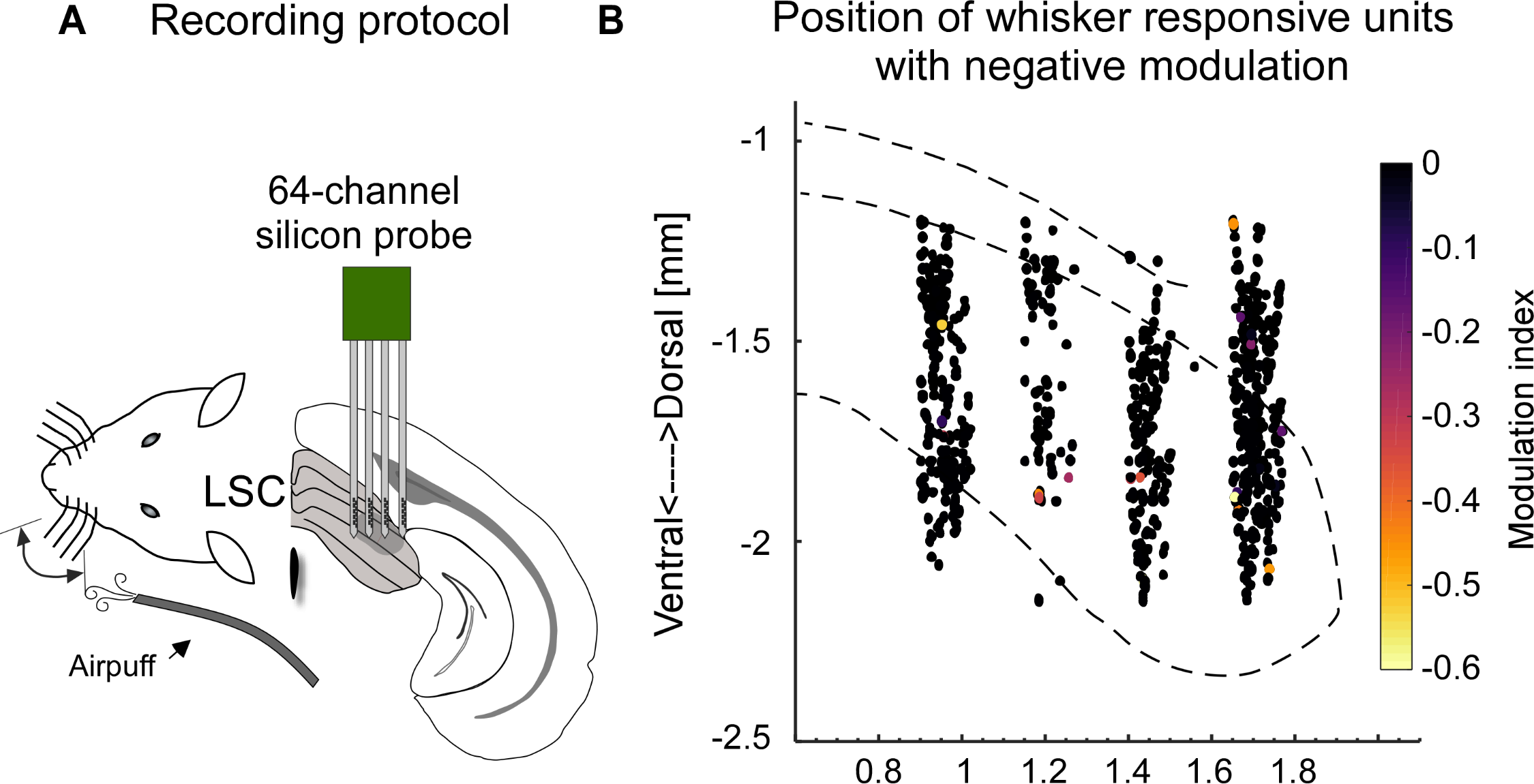

Supplement: S10 Fig — Related to Fig 2. (A) Experimental schematic of whisker airpuff stimulation and silicon probe recording in LSC in awake mice. (B) Summary from 12 recordings (1,005 units, 8 mice) mapped onto SC outlines through trilateration in CellExplorer [4,5]. Each dot depicts the location of a unit; colors indicate the negative modulation strength upon whisker stimulation (see Materials and methods). See main Fig 2C for modulated units. The data for S10B Fig can be found at: https://doi.org/10.11588/data/DNOSZG. LSC, lateral SC; SC, superior colliculus. (TIFF) [file pbio.3002126.s010.tiff]

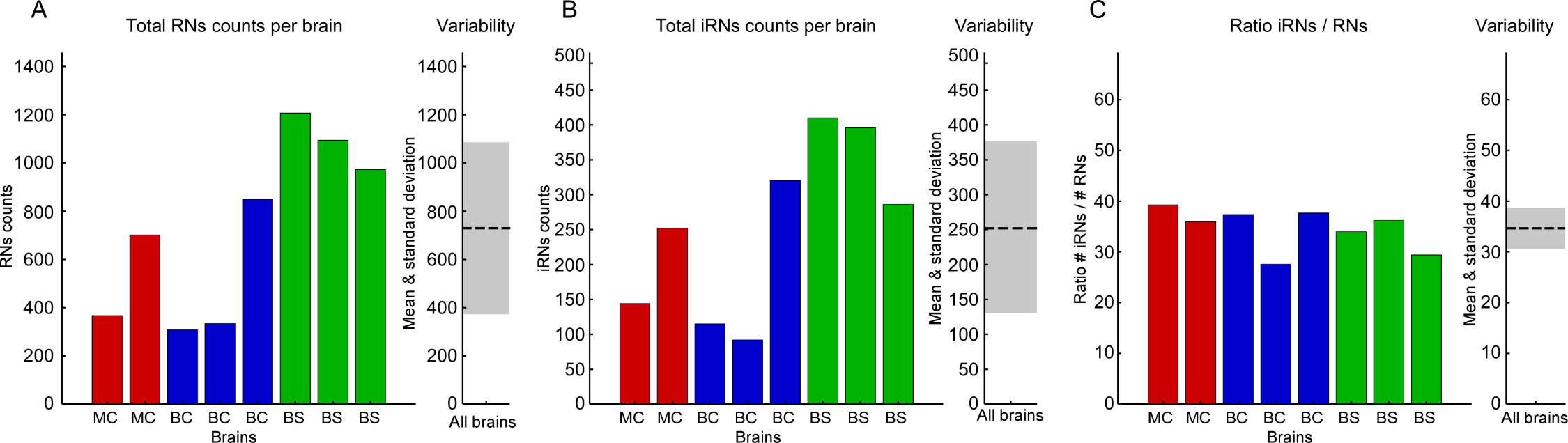

Supplement: S11 Fig — Related to Fig 5. (A) RN counts per brain per pathway (MC: red; BC: blue; Bs: red), mean (dashed line), and standard deviation (grey shaded area). (B) Same as in (A) but for iRNs. (C) Same as (A, B) but for the resulting ratio of iRNs/RNs. The data for S11A-S11C Fig can be found at: https://doi.org/10.11588/data/DNOSZG. BC, barrel cortex; Bs, brainstem; iRN, inhibitory RN; MC, motor cortex; RN, recipient neuron. (TIFF) [file pbio.3002126.s011.tiff]

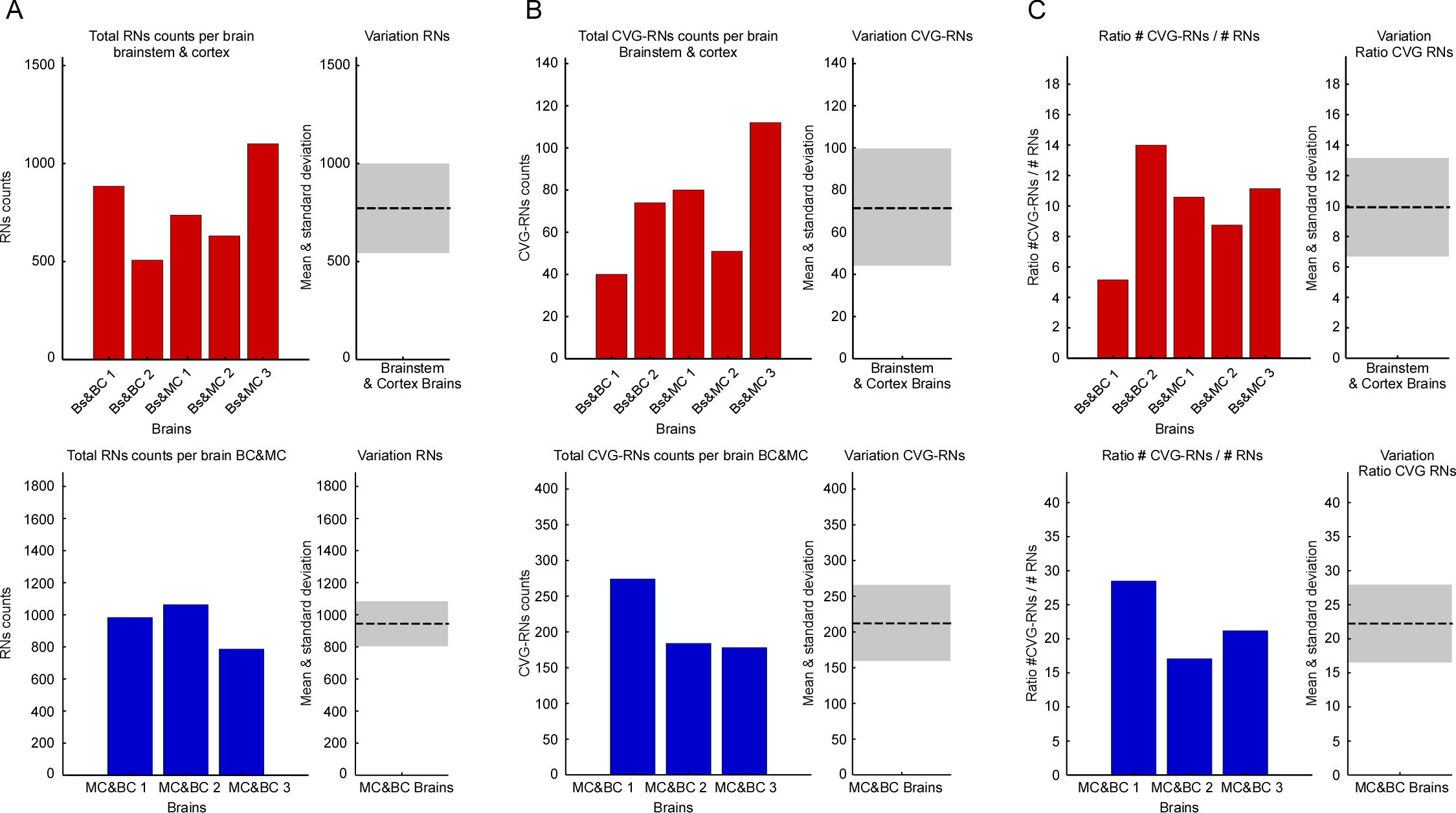

Supplement: S12 Fig — Related to Fig 6. (A) RN counts per brain per pathway (brainstem and cortex: upper panel, red; and MC and BC: bottom panel, blue), means (dashed lines), and standard deviations (grey shaded areas). (B) Same as in (A) but for CVG-RNs. (C) Same as (A, B) but for the resulting ratio of CVGs/RNs. The data for S12A-S12C Fig can be found at: https://doi.org/10.11588/data/DNOSZG. BC, barrel cortex; MC, motor cortex; RN, recipient neuron. (TIFF) [file pbio.3002126.s012.tiff]
