# Supplementary material for: Monosynaptic trans-collicular pathways link mouse whisker circuits to integrate somatosensory and motor cortical signals
Source: PLoS Biol. 2023 May 19;21(5):e3002126. doi: 10.1371/journal.pbio.3002126 (PMC10234540; doi:10.1371/journal.pbio.3002126)
Supplement: S1 Table — (DOCX) [file pbio.3002126.s013.docx]

| **Figure** | **Comparison** | **Statistical Test** | **P value =** |
| --- | --- | --- | --- |
| 4D | RNs Median location | Kruskal-Wallis | Chi Prob=7.8572e-06 |
|  | MC-RNs vs BC-RNs | post-hoc Bonferroni Method | 6.4351e-06 |
|  | MC-RNs vs Bs-RNs | post-hoc Bonferroni Method | 0.008951 |
|  | BC-RNs vs Bs-RNs | post-hoc Bonferroni Method | 0.061802 |
| 4F Histogram D-V | D-V Cortex vs brainstem Medians | Wilcoxon Rank sum | 0 |
| 4F Histogram M-L | M-L Cortex vs brainstem medians | Wilcoxon Rank sum | 0 |
| 5G | iRN median proportion per slice | Kruskal-Wallis | Chi prob=6.8211e-04 |
|  | MC-iRNs vs BC-iRNs | post-hoc Bonferroni Method | 1 |
|  | MC-iRNs vs Bs-iRNs | post-hoc Bonferroni Method | 1 |
|  | BC-iRNs vs iBs-RNs | post-hoc Bonferroni Method | 1 |
|  | MC-iRNs vs GABA | post-hoc Bonferroni Method | 0.0019154 |
|  | BC-iRNs vs GABA | post-hoc Bonferroni Method | 0.01571 |
|  | Bs-iRNs vs GABA | post-hoc Bonferroni Method | 0.0092727 |
| 6C | CVG-RNs median location | Kruskal-Wallis | Chi prob=8.1595e-10 |
|  | Bs&BC vs Bs&MC-RNs | post-hoc Bonferroni Method | 3.3295e-10 |
|  | Bs&BC vs BC&MC-RNs | post-hoc Bonferroni Method | 0.00024089 |
|  | Bs&MC vs BC&MC-RNs | post-hoc Bonferroni Method | 0.00047532 |
| 6E | CVG-RN median proportion per slice | Kruskal-Wallis | Chi prop=4.2556e-14 |
|  | Bs&BC vs Bs&MC-RNs | post-hoc Bonferroni Method | 1 |
|  | Bs&BC vs BC&MC-RNs | post-hoc Bonferroni Method | 2.8236e-08 |
|  | Bs&MC vs BC&MC-RNs | post-hoc Bonferroni Method | 1.1304e-12 |
| S2B | MC boutons vs BC boutons | Wilcoxon Rank sum | 0.0057 |

## S1 Table. Statistical comparisons per figure.
