# Supplementary material for: Monosynaptic trans-collicular pathways link mouse whisker circuits to integrate somatosensory and motor cortical signals
Source: PLoS Biol. 2023 May 19;21(5):e3002126. doi: 10.1371/journal.pbio.3002126 (PMC10234540; doi:10.1371/journal.pbio.3002126)
Supplement: S2 Table — (DOCX) [file pbio.3002126.s014.docx]

| **Experiment** | **Neuron Type** | **N (neuron counts)** |
| --- | --- | --- |
| Whisker responsive units Fig 2C and S10 Fig | Whisker responsive units/Recorded units | 325 / 1005 |
|  | Positive Modulation/Total whisker responsive | 302 / 325 |
|  | Negative modulation/Total whisker responsive | 23 / 325 |
| Retrograde depth profile Fig 3D | SC-projecting MC neurons | 71 |
|  | SC-projecting BC neurons | 55 |
| Recipient Neurons (RNs)  Fig 4D | MC-RNs | 5263 |
|  | BC-RNs | 2927 |
|  | Bs-RNs | 6702 |
| Cortical and brainstem inputs Fig 4F | Cortical RNs (MC & BC), Brainstem RNs | 1 brain |
| GABAergic Neurons Fig 5A | GABA (Gad-GFP), NeuN | 84, 384 |
| GABAergic Neurons Fig 5E | iRNs (Cre & Flpo), RNs (Flpo) | 41, 119 |
| GABAergic Neurons Fig 5G | GABA (GAD-GFP), NeuN | 300, 1296 (10 slices, 30 sample sections) |
| Inhibitory Recipient neurons iRNs, RN Fig 5G | MC-iRN (Cre &Flpo), MC-RN (Flpo) | 396, 1068 (24 slices) |
|  | BC-iRN (Cre &Flpo), BC-RN (Flpo) | 527, 1492 (34 slices) |
|  | Bs-iRN (Cre & Flpo), Bs-RN (Flpo) | 1092, 3274 (39 slices) |
| Convergent Neurons CVG Fig 6C | MC & BC-CVG-RN (Cre & Flpo) | 707 |
|  | Bs & MC-CVG-RN (Cre & Flpo) | 264 |
|  | Bs & BC-CVG-RN (Cre & Flpo) | 115 |
| Convergent Neurons CVG Fig 6C | Cortex & cortex vs  Cortex & brainstem | 52 slices  65 slices |
| Convergent Neurons CVG-RN Fig 6E | MC&BC-CVG-RN (Cre&Flpo), RN (Flpo) | 636, 2832 (55 slices) |
|  | Bs & MC-CVG-RN (Cre & Flpo), RN (Flpo) | 243, 2453 (63 slices) |
|  | Bs & BC-CVG-RN (Cre & Flpo), RN (Flpo) | 114, 1391(27 slices) |
| Gaussian mixture model Fig 6F | MC & BC-CVG-RN (Cre & Flpo) | 222 |
| Functional Convergence Fig 7C | Total Responsive units | 30 |
|  | MC-L5 → SC responsive units | 25 |
|  | BC-L5 → SC responsive units | 14 |
|  | MC & BC → SC responsive units | 9 |
| Bouton diameter S2B Fig | MC → SC boutons BC → SC boutons | 44  100 |
| Comparison variability iRNs Ratio  S11 Fig | MC-RNs [2 brains] BC-RNs [3 brains] BS-RNs [3 brains] MC-iRNs [2 brain] BC-iRNs [3 brains]  BS-iRNs [3 brains] | [307; 701] [308; 334; 850] [1207; 1094; 973] [144; 252] [115; 92; 320] [410; 396; 286] |
| Comparison Variability CVG-RNs S12 Fig | Brainstem & Cortex RNs  [5 brains]  Cortex & Cortex RN [3 brains]  Brainstem & Cortex CVG-RNs [5 brains]  Brainstem & Cortex CVG-RNs [3 brains] | [884; 507; 737; 631; 1101]   [983; 1063; 786]   [40; 74; 80; 51; 112]   [274; 184; 178] |

## S2 Table. Sample sizes per figure.
