# Supplementary material for: Monosynaptic trans-collicular pathways link mouse whisker circuits to integrate somatosensory and motor cortical signals
Source: PLoS Biol. 2023 May 19;21(5):e3002126. doi: 10.1371/journal.pbio.3002126 (PMC10234540; doi:10.1371/journal.pbio.3002126)
Supplement: S3 Table — Reported values are medians, Q1, and Q3. Related to Figs 2 and 7. (DOCX) [file pbio.3002126.s015.docx]

|  | **Spontaneous firing rate [Hz]** | **Evoked firing rate [Hz]** | **Onset latency [ms]** |
| --- | --- | --- | --- |
| **MC-L5 responsive units** | 1.26, 0.71, 2.98 | 111.10, 83.33, 142.36 | 12.68, 11.59, 13.56 |
| **BC-L5 responsive units** | 1.23, 0.96, 4.29 | 95.83, 70.83, 106.25 | 9.51, 8.74, 12.65 |
| **MC-L5 & BC-L5 responsive units** | 1.17, 0.90, 2.45 | MC-L5: 122.92, 78.64, 128.64  BC-L5: 95.83, 67.19, 111.63 | MC-L5: 12.24, 11.50, 13.34  BC-L5: 9.58, 8.73, 12.64 |
| **Whisker**  **responsive units** | 2.55, 1.0, 5.46 | 30.18, 14.59, 63.79 | 31.46, 25.08, 47.15 |

## S3 Table. SC unit population spiking characteristics. Reported values are medians, Q1, and Q3. Related to Figs. 2, 7.
