# Supplementary material for: Monosynaptic trans-collicular pathways link mouse whisker circuits to integrate somatosensory and motor cortical signals
Source: PLoS Biol. 2023 May 19;21(5):e3002126. doi: 10.1371/journal.pbio.3002126 (PMC10234540; doi:10.1371/journal.pbio.3002126)
Supplement: Attachment — Submitted filename: renamed_143d2.pdf [file pbio.3002126.s016.pdf]

REVIEWS:

Reviewer's Responses to Questions

Do you want your identity to be public for this peer review?

Reviewer #1: Yes: Ehud Ahissar

Reviewer #2: No

Reviewer #3: Yes: Wolfger von der Behrens

Reviewer #1: The SC is often thought to be an "old cortex", that is, it is thought that it used to contain many functions later recapitulated by the neocortex. As such, the SC is at the midst of many sensory-motor pathways, that is, pathways connecting sensory information to motor actions. Also, the SC is subject to rich cortical control, possibly mediating the necessary coordination between the "new" and "old" sensory-motor hubs.
The current study by Martin-Cortecero et al sheds novel light on this evolutionary dialogue. Focusing on the input to the SC, in fact to the whiskers-related part of the SC, the study revealed that cortical neurons directly control the output neurons of SC, via converging inputs from at least two cortical areas - motor cortex and barrel cortex. This control affects the brainstem-SC-brainstem sensory-motor arc, as well as the outputs from SC to midbrain stations.
The study presents micro-anatomical evidence for this convergence in the whisker tactile modality by revealing the input/output organization of the lateral zone of the superior colliculus (SC) that contains the whisker-related pathways. The authors show that cortical and trigeminal inputs target the GABAergic and non-GABAergic recipient neurons of this zone, which provide the output directed to diencephalic and brainstem nuclei - the former including "high-order" thalamic nuclei involved in the processing of self-motion (PO, ZI, PF and VM) and the latter involving nuclei involved in the execution of self-motion (PnC, Gi and 7N).
The methodological basis of this study includes modern methods to achieve the goals set in the introduction. The authors used multiple fluorescent tracing strategies with a combination of classic tract-tracing and viral tracing methods. They mapped the input-output connectivity of the mouse SC on the single-cell level with transsynaptic tracing, used intersectional viral approaches and optogenetic-assisted electrophysiology, applied computational neuroanatomical methods to analyze the representative cortico-tectal pathways that span the rostral-to-caudal and medial-to-lateral extent of the cerebral cortex and SC. Obtained labels were overlapped by the respective atlas templates.
This study is timely and exciting - it reveals information that is crucial for understanding the operation and coordination among parallel sensory-motor arcs - those running primarily via the SC and their evolutionary competitors running via the neocortex. The conceptual basis of this work, the outlines of the approach, methods and analyses are appropriate for the objectives of the study. The MS is clearly and scholarly written.
There are several issues that require the attention of the authors.
Thank you for your in-depth and scholarly review and the helpful suggestions to better frame our paper. We are especially grateful for the specific recommendations for phrasing and we took the liberty of adopting several of these into the revised paper.

Major:
The authors do not refer to the parallel-arcs context mentioned above, relating the SC-mediated and the neocortex-mediated sensory-motor arcs to each other. As a result, the paper lacks an important angle that seems to be crucial for understanding the meaning of their results. I thus strongly suggest to add it, at the outset, and to add interpretations of the data in that direction.
We agree on this point, especially that linking our results to parallel sensory-motor arcs emphasizes the importance of this study in a broader context. We implemented this framework into critical places throughout the paper, in the abstract, intro and discussion.

Two terminology/phrasing issues refer to the above:
a) sensorimotor. This term (or equivalently "sensory-motor") typically refers to the sensory-motor arc. That is, to the processing done in brain circuits eventually transfroming activity from the sensory end to the motor end. Here the authors use "sensorimotor" when referring to convergence of signals from sensory and motor cortices. This is confusing and I suggest to stick to the traditional usage of this term. Of course, traditions are in the eye of the beholder, thus if the authors do not agree with the above they do not have to adopt this recommendation. In any case, it should be noted in the paper that layer 5 neurons of both S1 and M1 convey motor-related signals.
Thank you for pointing this out. We clarified this ambiguity, by using more specific phrasing (i.e. instead of sensorimotor convergence, we now specify "convergence between sensory and motor cortex in SC" or "Single LSC units integrate sensory- and motor cortex inputs."). We also added the original sensory-motor definition in the second sentence of the introduction. Furthermore, we included the suggestion to discuss that layer 5 neurons of both S1 and M1 convey motor-related signals (see revised discussion).

b) upstream/downstream (e.g., Abstract: "The results …by which upstream neurons in motor- and somatosensory cortices are linked to specific downstream whisker circuits in the brainstem …") - the "upstream" and "downstream" affiliations imply some agreement about the direction of information/processing flow among these stations. However, this doesn't seem to be the case. I suggest a different terminology, respecting the complex interplay between the sensory-motor arcs and the loopy organization of processing (both between the arcs and between the sensory and motor ends).
We followed the suggestion and now avoid the upstream-downstream terminology wherever appropriate. In some places we still use the term "downstream" when we specifically refer to SC targets in the context of the cortex->SC->target axis.

And one additional suggestion:
c) one possible distinguishing function between SC- and cortex-related arcs is the primary focus of their corresponding brain-world loops - self-motion for SC-mediated loops and external objects for cortical-mediated loops. If the current results can be interpreted in a way supporting or disproving such distinction it is strongly suggested to add such interpretations. In any case - the distinction is worth mentioning.
Thank you for this stimulating thought. We now discuss this framework in the completely revamped concluding paragraph in the discussion. In brief, the idea of the differential focus of the two systems on self-motion for SC-mediated loops and external objects for cortical-mediated loops makes sense in the context of our results. One possible implication however that could be drawn from this division would be that SC-initiated self-motion operations would always need to rely on information from the cortex about external objects. This is an interesting idea, which would be really great to investigate in a review or experimentally. In parallel to the suggested framework, we also propose a related framework in the discussion, in which SC can perform low-level sensorimotor actions

principally without the cortex. The cortex however can interfere with these SC sensorimotor actions, by remodelling the balance between excitatory and inhibitory trans-collicular pathways. This would endow the mammalian brain with more flexibility compared to brains in which the SC is the sole processing circuit for sensorimotor functions.

Other comments

1) It may be useful if the authors designate the zone- and layer-specific nomenclature within the SC in their figures to facilitate more detailed referencing and precise quantification of the revealed inputs. The authors touched these issues when defining the three principal questions about the organization of transcollicular pathways. In setting out the first question (P.2, 2nd para), the authors referred to the problem of the zonality of the distribution of input structures in the SC when citing the article by Benavidez et al. (2021) and calling them "mesoscopic modules" that include the structures identified in the current study. It looks like the authors do not reject their significance, which allows the use of such nomenclature in this study.

Thank you for the suggestion to compare our results on the organization of recipient zones to the radial input zones from Benavidez et al. (2021). To implement this comment, we overlaid the recipient zones from MC, BC and Bs to the approximate outlines of the radial zones determined by Benavidez et al (2021). Since Benavidez et al. (2021) estimated polar coordinates of recipient zones with different approaches than ours, the comparison between our and their structures can only be an estimate. Nevertheless, the comparison shows that our recipient zones fall into the Benavidez' radial zones as follows: BS → lateral / centrolateral; BC → centrolateral; MC → centrolateral/centromedial. We now show this data in the revised Fig. 4E and describe the comparison in the corresponding results text. To further facilitate comparison of our results to previous SC literature, we also show an example of RNs registered to the SC Latin layer terminology in Supplementary Fig. S3.

2) In the subsection "Slicing and immunolabeling" (P. 31), the authors wrote that the brain was "postfixed for 12 h in 4% PFA at 4°C". It looks as not enough to prevent freezing damage of the tissue and to preserve its structural integrity during slicing. In the studies that use similar methods, effective postfixation of the mouse brain is achieved by using hypertonic sucrose [see, for example, papers by Wall et al. (2010) and Castro-Alamancos and Favero (2016)], or by postfixation in 4% PFA for 24-48 h at 4°C (Benavidez et al., 2021). If the authors used sucrose or other cryoprotectants in this study, it should be mentioned.

The reviewer is right that we did not use cryoprotectant. The use of cryoprotectant was not necessary in our study because we kept our samples at 4°C or above, without freezing at any point in the process. Thus, we would not expect and we did not observe freezing damage for the tissue that was used in this study because it was never frozen. We checked two studies mentioned by the reviewer: Benavidez et al., 2021 froze their samples at -20°C after slicing, similarly Castro-Alamancos and Favero (2016) resectioned their slices on a cryostat. For these procedures cryoprotectant is indeed necessary but since we did not use any procedures involving freezing we did not use cryoprotectant.

3) In figure 3E, the spatial position of the atlas templates needs to be corrected with respect to the midline and the scale.

Thank you for this suggestion. We revised Fig. 3E accordingly.

4) The study of the LSC projection neurons in Sp5o looks quite reasonable because of its involvement into whisker circuitry which was previously revealed (Erzurumlu et al. 2010; Ebert et al., 2021). However, as it was shown 40 years ago by Huerta et al. (1983), other trigeminal nuclei, such as Principal and Interpolaris, have a larger number of neurons projecting to the SC.

If the authors have the data about neuronal populations of other brainstem nuclei that project to the SC, it would be interesting to compare them with the Sp5o.
In the brainstem we observed the strongest and clearest somatic labelling of LSC projecting neurons in Sp5. Within Sp5 we observed most of the signals in the oralis part and some in the interpolaris. We also found a few somata in the adjacent parvocellular reticular nucleus alpha portion (PCRtA, see revised Fig. 3E). We also closely checked again in other rostrocaudal sections. Some signal was indeed seen in PrV, however in contrast to the Sp5, the signal in the PrV was clearly axonal and not somatic and stems most likely from PrV-projecting L5 neurons (in somatosensory and/or motor cortex), which are retrogradely labelled via the LSC injection.

5) The MS contains a lot of standard and non-standard abbreviations. An abbreviation should appear at the first occurrence of the word, which is not always respected by the authors. For example, the abbreviation for zona incerta (ZI) appeared on P. 17, while these unabbreviated words are seen on P. 3 (3rd line from the bottom).
Thank you, we corrected this.

6) In the third horizontal row, fourth vertical cell of the Table 1: replace "mCHerry" by "mCherry".
Thank you, we corrected this.

7) P. 7, Line 7 from the top: "Exact N in Supplementary Table S2." should be moved to the Line 3 of P.7 because it is related to the legend for Fig. 3D, as it is mentioned in the Table S2.
Thank you, we corrected this.

8) The term "anterior-posterior" is used in the MS to designate coordinates and axes in many places in the text, in figures and figure captions and in Table 2. It should be replaced by the term "rostro-caudal", referring to the coordinates of the rodent brain.
We replaced "anterior-posterior" with "rostro-caudal" throughout the paper.

Reviewer #2: The manuscript by Martin-Cortecero et al. entitled 'Monosynaptic trans-collicular pathways for sensory-motor integration in the whisker system' is thorough and relevant. The authors describe monosynaptic transcollicular pathways connecting motor and somatosensory cortices, as well as the trigeminal complex, with thalamus and zona incerta via long range excitatory and inhibitory connections. The novelty of the manuscript strives in the description of monosynaptic, somatosensory, GABAergic pathways, as well as the convergence of motor and sensory terminals on superior collicular projection neurons. The experiments are thorough and convincing. The manuscript is well written. The work reveals a novel loop of cortico-collicular-thalamic projections where sensory-motor information can be integrated. In addition it reveals a novel loop of cortico-collicular-zona incerta projections that is very likely to play an important role in the modulation of sensory-motor interactions.
We thank the reviewer for the detailed review and constructive comments for improving our manuscript.

Major comments:

My main concern is with the quantifications of anatomical distribution of projections and percentage of iRN and CVG-RN. I would need to be convinced that these quantifications are not biased by the viral injection location or the efficiency of the different viruses. Comparing results across mice and/or using different viruses would make the case stronger. Given the number and

strength of experiments reported here, it might be enough to justify the value and report it as indicative.

Thank you for pointing out this potential caveat, which we can clarify. As suggested by the reviewer, we more explicitly state in the text that the numbers are estimates. In addition we thoroughly indicate the variation of all measurements (Figs. 5 F,G; 6C-F).

We would like to emphasize the following points and additional analysis in regards to the quantifications:

1: To minimize potential bias stemming from variations in the injection parameters, we calculated the iRN and CVG-RN percentages based on ratios between iRNs/RNs and CVG-RNs/RNs. Normalizing the iRN and CVG-RN counts to the total RN counts in each animal should reduce injection parameter bias, such that variations in the RN numbers between animals due to differences in the viral injection location, will not lead to systematic errors in the ratios. Importantly, we do not draw any conclusions on the absolute numbers but only on the ratios.

2: To better quantify the variability of the data and motivated by the reviewer's comment we conducted additional variability analysis and included these together with the raw counts for each experiment and brain into the paper. The variability of the raw counts and resulting ratios were comparable to each other. For the iRN calculations, the variability of the ratios was even substantially smaller compared to the raw counts, showing that these ratio measurements are stable (within boundaries), despite variations of the RN numbers across individual animals (new figures S11 and S12).

3: The CVG estimates based on the anatomical analysis (Fig. 6) are comparable to the functional convergence measures (Fig. 7).

4: We provide new data in which we tested how much the injection location of the reporter into SC affects the labelling of RNs. In brief, we injected two reporter viruses with different colours (red and green) into adjacent locations within the SC and analysed the resulting labelling. We find that RNs are confined to specific locations in SC (RN-zones) and very few RNs were labelled by the second reporter outside of the RN zone. This demonstrates that our injection parameters to label RNs largely encompassed the whole RN zone, minimizing the chances that reported numbers are biased by missing RNs in these experiments. We provide this data in the new Supplementary Fig. S9.

In summary, we thank the reviewer for this comment and we now refer to these calculations as estimates as suggested and provide additional analysis for the validation of the approaches and ratio calculations as well as the underlying raw data.

Minor comments aiming to help with the clarity of the manuscript:

Title: Short titles are nice but, since S1 projects also to inferior colliculus and inferior colliculus also perform sensory-motor integration, this title is ambiguous.

We agree that the term "collicular" includes the inferior colliculus. Referring to the colliculus as general structure in the title seems appropriate and common for broad readership (for example "cortical", "collicular", "thalamic") and is also common in recent studies on the SC, see for example e.g. (Beltramo & Scanziani, 2019; Campagner et al., 2023). In addition, we define SC in the first sentence in the abstract, to prevent ambiguity. We would therefore like to keep the title, if the reviewers and editors agree.

Abstract: 'we mapped the anatomical and functional…'. Not sure what it is meant by mapping the functional input-output properties since no function was assessed. Maybe more accurate to say 'physiological'?

Thank you for pointing this out. We changed the sentence accordingly, which now reads: "Here, we mapped the connectivity of the whisker-sensitive region of the SC in mice with trans-synaptic and intersectional tracing tools and in vivo electrophysiology."

Abstract: '…GABAergic neurons, which in turn give rise to hitherto unknown…' This sentence gives them impression that all GABAergic RN lead to long-rang connections. I would rephrase.
Thank you, we changed the sentence accordingly which now reads: "More than a third of the cortical recipient neurons in the whisker SC are GABAergic neurons, which include a hitherto unknown population of GABAergic projection neurons targeting thalamic nuclei and the zona incerta."

Introduction: while the idea of focusing on specific question and describing them in turn is very good, I would briefly introduce the same questions in the first paragraph of the introduction.
Thank you for this suggestion. We introduce the three specific questions in the first paragraph of the introduction. The following three paragraphs now pick up these three questions in the beginning of each paragraph. We also slightly changed the order of the first mentioning of the questions to match the order of the following paragraphs.

Consistency in naming: e.g. in the first paragraph of the introduction the question is about 'target in the diencephalon and brainstem, while in the first question t refers to projections to 'thalamus and brainstem'. Both are correct but maybe for the general reader it is worth including more details? For example to say first 'diencephalon (including thalamus)', and then use 'diencephalon' both times?.
Thank you, we adopted this suggestion. Diencephalon is now used homogeneously and the sentence in the first paragraph now reads: "More specifically, it is not well understood how cortico-collicular pathways engage with SC microcircuitry and in turn generate SC output signals to the brainstem and diencephalon (including thalamus)."

Introduction, question 2. Describe visual and somatosensory SC for a wider PloS Biology audience
Thank you, we implemented this suggestion and now describe these two terms in the introduction: "SC circuits have mostly been studied in relation to vision [2,6–8], i.e. looking at functions of the 'visual SC'. In contrast, somatosensory functions of the SC are less studied and less understood, even though somatosensory functions such as whisker sensation are vital for rodents and other animals [9–12]. This study set out to address the involvement of 'somatosensory SC' circuits in the whisker system and focused on three principal questions about the organization of trans-collicular pathways that mediate cortical input to SC downstream targets (Fig. 1). "

Page 3, last paragraph: Is 'intersectional' a standard term? I am not sure what it means? Across SC sections? Histology?
We adopted this term from the literature in which it is used to describe the strategies to characterize intersections between overlapping cell populations. For example in our study, the recipient neurons (RNs) contain a subpopulation of inhibitory neurons (iRNs). The approach we used is based on the differential labelling of neurons which fulfil different conditions. For RNs to be labelled, they need to receive input from an area of interest (e.g. BC). For the iRNs to be labelled they need to fulfil both, the previous condition, AND the additional condition to express cre. The intersection of these two conditions represent iRNs and the whole population represent RNs. This term can be considered a standard term in the context of genetic intersections by means of adeno-associated virus or site-specific recombinase systems or CRISPR technologies to define specific cellular subpopulations; here are few examples for studies which use this term in a similar context as our (Fenno et al., 2014; Hafner et al., 2019; Kitanishi et al., 2022; Poulin et al., 2018; Weinholtz & Castle, 2021). We added a new paragraph to the methods "Trans-synaptic anterograde and intersectional labelling" which explains the term and strategies in more detail.

Page 3, last paragraph: 'optogenetically-assisted electrophysiology', maybe use the more common term opto-tagging?
Thank you for bringing this up. The term optotagging is indeed commonly used, however it usually refers to the “tagging” of ChR2-expressing neurons, which was not the case in our study. In our study, the purpose of optogenetic stimulation was to identify monosynaptic connections between BC/MC neurons and recipient SC neurons (measured as postsynaptic responses with appropriate latencies). In addition to the different purposes, the methodologies are also slightly different. In optotagging experiments, the electrode and laser are in the same place, while in our experiments we placed the laser into MC and BC and the electrode into SC. Because of the different purpose and method of optotagging, the term might thus be misleading in the context of the present study. Nevertheless, we sharpened the sentence which now reads: “optogenetically assisted connectivity quantifications in vivo”.

Page 3, last paragraph: the sentence 'suggesting trans-collicular circuits for cortical disinhibitory control of higher-order thalamus' is loaded with very specific information that has not ben previously mentioned. Maybe a bit of a mouthful for a general conclusion at the end of the introduction?
Thank you for pointing this out. We simplified this part.

Figure 2A: like all the others schemes in this manuscript with the exception of those in Figure 1, this scheme is beautiful and very useful but very small!!! Maybe have the detailed scheme once enlarged and from then on a less realistic but larger scheme for MC, BC and Bs. For example by Figure 5A, we know what SC looks like and where it is and it would suffice to show just SC.
We made the suggested changes. Fig. 2A: size increased. Figs. 5A, E: scheme now shows enlarged view of SC.

The same is true for most of the neuron target reconstructions, e.g. Fig 5D, 6B, 8A. One could cut the hippocampus and overlap the sections slightly.
We implemented the suggestion in all RN reconstruction figures.

Page 4, results, 1st paragraph: it would be useful to have the information on how many mice were used together with that of the number of recordings.
This information is now added to the results and figure caption.

Figure 2C: I don't see the separation between positive and negative modulation referred to at the beginning of the results.
The reviewer is correct, Fig. 2C shows only positively modulated units. Negatively modulated units constituted a minority of all whisker modulated units (23/325). We further clarified this in the text and legend and also added a new Supplementary Fig. S10, which shows the negatively modulated units. Thank you for pointing this out.

Figure 2B shows a clear bimodal distribution between fast and slow responses. All of them look positively modulated by the whisker at some point? Please explain in more detail in the text.
The reviewer is correct, the whisker responses are bimodal. These fast and slow components have been described before in anesthetized mice and rats and were robustly seen in our awake mouse recordings. Castro-Alamanco’s work suggest that the fast component is due to trigeminal input (“trigeminotectal”), while the slow component is due to input from barrel cortex (“corticotectal”) (Castro-Alamancos & Favero, 2016). As suggested, we describe the bimodality now in the results (first paragraph).

Page 4, last sentence: I get the meaning but I think the sentence needs rephrasing.
Thank you, we edited this section for clarity.
Old:
“To do so, we estimated stereotaxic coordinates from the recordings to targeted retrograde virus (rAAV-ChR2-tdTomato, [28,29]) which retrogradely labelled upstream LSC-projecting neurons (Fig. 3A). LSC-projecting neurons were found in multiple cortical and subcortical areas (Fig. 3B), …”
New:
“To do so, we targeted a retrograde virus (rAAV-ChR2-tdTomato, [29,30], Fig. 3A) to the whisker-sensitive region in LSC, based on stereotaxic coordinates estimated from the recordings (Fig. 2). LSC-projecting neurons were found in multiple cortical and subcortical areas (Fig. 3B),...”

Page 7: Bs-projecting. I think this is the first time the abbreviation Bs is used other than the figure.
The abbreviations MC, BC and Bs are introduced together in the first results section in which we describe the inputs to LSC. We now slightly changed the order of the sentence, so that these definitions better stand out.

Page 7, last paragraph. I am not sure I understand the new conclusion here? The tracing has ben done in the LSC, which is already identified as somatosensory. Do the authors refer to 'whisker SC' as a specific form of somatosensory?
Yes, that is correct. To our knowledge there is no data available to make the conclusion that the LSC as a whole is exclusive for whisker signals. We provide multiple lines of evidence (anterograde, retrograde anatomy, boutons, whisker responses) that the “whisker SC” is located in the LSC, however we don't think it is accurate to state that the LSC is equal to the whisker-SC. To the best of our knowledge there is no comparable study in the literature that was able to combine anatomical, connectivity and functional evidences to pinpoint the identity of a whisker SC and thus we believe that this conclusion of our study should be emphasized as a distinct conclusion.

Figure 4C and E do not seem to match well. In the Bs example in Figure 4c many neurons seem to be in the ventral layer.
The reviewer is correct, that Bs-RNs in 4C are located more ventrally. However, we think there was a misinterpretation of Fig. 4E, as Bs-RNs are also located in the ventral regions compared to MC-RNs and BC RNs. Please also compare to Fig. 4F in which we analysed the distribution of Bs-RNs along the dorso-ventral axis, which similarly shows that Bs-RNs are located more ventrally than the cortical RNs. Moreover, Fig. 4E also shows overlap among recipient regions in more dorsal areas, consistent with convergence RNs in figure 6A and 6B.

Figure 4F; please include in the legend how many mice were used for co-labelling.
We added the information to Fig. 4F and to Table S2, that one animal was used for co-labeling cortical (MC and BC) RNs and Bs-RNs neurons.

Page 9: the term trigemino-collicular is used in the titles and legends, but the term brainstem and abbreviaton Bs are used in most of the text. Is there a reason not to chose one?
We homogenized the paper to “brainstem”, wherever appropriate.

Figure 5A, 22% of cells are reported as being GABAergic, but in the text it says 23%. Pleas round consistently.

Fig. 5A shows the quantification of a single section as an example for the methodology. In this example the proportion was ~22% In Fig. 5f-G and in the remaining paper we show and refer to the quantification of the full sample (n=30 sections from 10 slices, 1 brain), which is ~23%. This was not clearly indicated and is now fixed, thank you.

Discussion, paragr. 2: 'midbrain and brainstem'. I assume that, if the focus is on to targets, 'diencephalon and brainstem' are meant?
Yes thank you, we correct this.

Discussion, paragr. 2: '…fundamentally different from that found in other layered circuits - for example, compared to the canonical circuit scheme of the cortex, in which layer 4 neurons…'. I am not 'fundamentally different' is the right word. If the example would have been layer 6, the pattern wouldn't look so different. The long-range inhibitory projections are unusual but maybe not fundamentally different (see, as an example, the work on long-range inhibitory projections form the ZI to cortex by Schoeder et al. from the Letzkus lab in BioRxiv)
Thank you. We removed "fundamentally". However, we would like to emphasize that this statement explicitly refers to the canonical circuits scheme of cortical processing, in which L4 is regarded as the main input and L6 as output. Within this prominent framework (may it require adjustment or not), the trans-collicular motif would be fundamentally different, in that SC input cells are at the same time output cells, while in the canonical circuits scheme of cortical processing, input cells and output cells are segregated. We believe that this is a central finding, with significant implications for collicular computations.

Reviewer #3: Martín-Cortecero and colleagues present a new pathway for whisker sensory-motor integration where long-range motor-sensory innervate in the lateral superior colliculus and from there inhibitory and non-inhibitory pathways target thalamic and brainstem nuclei.
This microanatomical study is very well performed and presents a somatosensory-motor circuit that has not been described before. The authors performed very well controlled tracing experiments by combining different viral systems for anterograde and retrograde labelling of neurons and their targets. They complement these experiments with awake recordings and optogenetic stimulation to demonstrate the functionality of the described circuits. The amount of data presented and the level detail is impressive and have to admit that I find it tremendously difficult to spot a weakness in this study or things to improve.
I consider this study to be relevant for a broader readership as the described trans-collicular circuits are likely performing very critical sensory-motor integration that runs in parallel to the well studies and described thalamo-cortical loop. While present study was performed in the whisker system, there is good reason to assume that it is not limited to this modality but may be more generally relevant for other (multimodal) sensory-motor integration functions as well.
Given the relevance and quality of the study I recommend it for publication without any reservations. However, I would like to urge the authors as a minor revision to make the full dataset including the raw data available through an appropriate repository such as Zenodo or similar.
We thank the reviewer for the encouraging review and the support for timely publication of the results, which we highly appreciate. We followed the suggestion to make the dataset available. We provide more raw data in the supplementary section (see new figures and new content in the tables), we also provide anatomical data and electrophysiology data in the public repository heiDATA: https://doi.org/10.11588/data/DNOSZG, and referenced the repository throughout the paper. For the electrophysiology data, we chose to use the emerging standard for neurophysiology time-series data, Neurodata Without Borders (NWB), and as such should be easily accessible and

reusable for the broader research community. Upon publication, these data will further be uploaded to the NWB-specific data repository DANDI.

References

Beltramo, R., & Scanziani, M. (2019). A collicular visual cortex: Neocortical space for an ancient midbrain visual structure. *Science*, *363*(6422), 64–69. https://doi.org/10.1126/science.aau7052

Campagner, D., Vale, R., Tan, Y. L., Iordanidou, P., Pavón Arocas, O., Claudi, F., Stempel, A. V., Keshavarzi, S., Petersen, R. S., Margrie, T. W., & Branco, T. (2023). A cortico-collicular circuit for orienting to shelter during escape. *Nature*, *613*(7942), 111–119. https://doi.org/10.1038/s41586-022-05553-9

Castro-Alamancos, M. A., & Favero, M. (2016). Whisker-related afferents in superior colliculus. *Journal of Neurophysiology*, *115*(5), 2265–2279. https://doi.org/10.1152/jn.00028.2016

Fenno, L. E., Mattis, J., Ramakrishnan, C., Hyun, M., Lee, S. Y., He, M., Tucciarone, J., Selimbeyoglu, A., Berndt, A., Grosenick, L., Zalocusky, K. A., Bernstein, H., Swanson, H., Perry, C., Diester, I., Boyce, F. M., Bass, C. E., Neve, R., Huang, Z. J., & Deisseroth, K. (2014). Targeting cells with single vectors using multiple-feature Boolean logic. *Nature Methods*, *11*(7), 763–772. https://doi.org/10.1038/nmeth.2996

Hafner, G., Witte, M., Guy, J., Subhashini, N., Fenno, L. E., Ramakrishnan, C., Kim, Y. S., Deisseroth, K., Callaway, E. M., Oberhuber, M., Conzelmann, K.-K., & Staiger, J. F. (2019). Mapping Brain-Wide Afferent Inputs of Parvalbumin-Expressing GABAergic Neurons in Barrel Cortex Reveals Local and Long-Range Circuit Motifs. *Cell Reports*, *28*(13), 3450–3461.e8. https://doi.org/10.1016/j.celrep.2019.08.064

Kitanishi, T., Tashiro, M., Kitanishi, N., & Mizuseki, K. (2022). Intersectional, anterograde transsynaptic targeting of neurons receiving monosynaptic inputs from two upstream regions. *Communications Biology*, *5*(1), 149. https://doi.org/10.1038/s42003-022-03096-3

Poulin, J.-F., Caronia, G., Hofer, C., Cui, Q., Helm, B., Ramakrishnan, C., Chan, C. S.,

Dombeck, D. A., Deisseroth, K., & Awatramani, R. (2018). Mapping projections of molecularly defined dopamine neuron subtypes using intersectional genetic approaches. *Nature Neuroscience*, *21*(9), 1260–1271. https://doi.org/10.1038/s41593-018-0203-4

Weinholtz, C. A., & Castle, M. J. (2021). Intersectional targeting of defined neural circuits by adeno-associated virus vectors. *Journal of Neuroscience Research*, *99*(4), 981–990. https://doi.org/10.1002/jnr.24774
